# Seborrheic Dermatitis Revisited: Pathophysiology, Diagnosis, and Emerging Therapies—A Narrative Review

**DOI:** 10.3390/biomedicines13102458

**Published:** 2025-10-09

**Authors:** Francisco José Navarro Triviño, Juan Pablo Velasco Amador, Irene Rivera Ruiz

**Affiliations:** 1 Department of Dermatology, Hospital Universitario San Cecilio, 18007 Granada, Spain; pablova.correo@gmail.com; 2 Instituto de Investigación Biosanitaria de Granada (ibs.GRANADA), 18012 Granada, Spain; 3 Department of Dermatology, Fundación Jiménez Díaz University Hospital, 28040 Madrid, Spain; riveraruizirene@gmail.com; 4 Inflammatory Immune-Mediated Chronic Skin Diseases Laboratory, Instituto Maimónides de Investigación Biomédica de Córdoba (IMIBIC), Reina Sofia University Hospital, University of Cordoba, 14004 Cordoba, Spain

**Keywords:** seborrheic dermatitis, inflammatory skin diseases, malassezia, skin microbiome, barrier dysfunction, JAK inhibitors, PDE4 inhibitors, biological therapies, facial dermatoses, non-invasive diagnostics

## Abstract

**Background**: Seborrheic dermatitis (SD) is a chronic, recurrent inflammatory dermatosis that primarily affects seborrheic areas such as the scalp, face, and upper trunk. Its etiology is multifactorial, involving sebaceous gland activity, immune dysregulation, skin barrier dysfunction, and alterations in the microbiome, particularly an overgrowth of *Malassezia* spp. **Objective**: This review provides an updated overview of the pathophysiological mechanisms of seborrheic dermatitis and critically examines current therapies and emerging treatments. **Methods**: A narrative review of the recent literature was conducted, including preclinical studies, clinical trials, and real-world evidence regarding SD pathogenesis and therapy. Special attention was paid to molecular pathways, microbiome-modulating strategies, and novel therapeutic agents. Results: Advances in transcriptomic and microbiome profiling have revealed a complex immunoinflammatory environment in SD, involving predominantly Th1, Th17, and Th22 axes. Conventional therapies are mainly based on antifungals, topical corticosteroids, and calcineurin inhibitors. However, new therapeutic approaches are under investigation, including PDE4 inhibitors (roflumilast, crisaborole, and apremilast), topical and oral JAK inhibitors, probiotics, and microbiome-targeted therapies. These agents offer promising results in selected patients, particularly those with refractory disease or facial involvement. **Conclusions**: SD remains a challenging condition due to its relapsing course and limited long-term therapeutic options. Emerging therapies represent a valuable opportunity to address unmet clinical needs, particularly in patients with severe, recurrent, or treatment-resistant forms.

## 1. Introduction

Seborrheic dermatitis (SD) is a chronic inflammatory dermatosis that most commonly affects the scalp, face, and upper trunk, areas with a high density of sebaceous glands [1]. The global prevalence of seborrheic dermatitis (SD) is estimated at 4.38% (95% CI, 3.58–5.17%), with higher rates observed in adults (5.64%) compared to children (3.70%) and neonates (0.23%) [2]. There is considerable geographical variation, with the highest prevalence reported in South Africa (8.82%) and the lowest in India (2.62%) [2]. Among individuals living with HIV, SD affects 20% to 83% of patients, particularly those with advanced immunosuppression or AIDS, and is often regarded as a clinical marker of immune status [3]. In patients with Parkinson’s disease, the prevalence ranges from 52% to 59%, with seborrheic dermatitis reported in over half of cases [4,5].

Clinically, SD is characterized by erythematous plaques covered with yellowish or whitish scales, often associated with pruritus. Although considered a benign condition, its chronic, relapsing course and visibility in exposed areas result in a significant emotional and functional impact [6]. Patients with SD are at increased risk for anxiety, depression, low self-esteem, and social impairment. The quality-of-life burden has historically been underestimated despite its clinical relevance [7].

The pathophysiology of SD remains incompletely understood. Several contributing factors have been identified, including seborrhea, cutaneous microbiome dysbiosis (particularly overgrowth of *Malassezia* spp.), epidermal barrier dysfunction, and an altered immune response involving Th1, Th17, Th2, and Th22 pathways. In recent years, non-invasive technologies such as tape stripping have enabled transcriptomic profiling of SD, opening the door to molecular biomarkers [8,9].

Current treatment strategies are mainly based on topical antifungals, corticosteroids, and calcineurin inhibitors. However, these strategies generally achieve only partial symptom control and are frequently followed by relapses. The absence of targeted therapies and the limited evidence available for specific patient subgroups highlight important unmet needs [10,11].

This narrative review was based on a comprehensive literature search in PubMed, Scopus, and Web of Science databases, covering studies published between 2000 and 2025. The search strategy included the following MeSH terms and keywords: “*Seborrheic Dermatitis*”*[MeSH]*, “*Malassezia*”*[MeSH]*, “*Skin Microbiome*”*[MeSH]*, “*Pathophysiology*”, “*Diagnosis*”, and “*Therapeutics*”. Additional articles were identified through manual reference screening. 

In this context, the present manuscript provides an updated review of the pathophysiology, diagnostic advances, and current and emerging therapeutic options in seborrheic dermatitis, with an integrated and evidence-based perspective.

## 2. Clinical Presentation

Seborrheic dermatitis occurs more frequently in men than in women. A higher incidence has also been reported among African American patients compared to white or Asian populations [2]. The disease follows a bimodal distribution, with a first peak in infancy and a second in adulthood, particularly among young adults [12].

Clinically, SD presents as erythematous-to-orange patches with thick, yellowish, greasy scales. Pruritus is the most frequent symptom, with variable intensity among patients, and is most commonly reported on the scalp. Some authors consider dandruff a non-inflammatory form of SD, characterized by fine white scales without underlying erythema [13].

Clinical manifestations may be typical or atypical, with considerable interindividual variability that clinicians must recognize [14]. Table 1 summarizes the main clinical features of SD by age group. The differential diagnosis is broad and can represent a clinical challenge in certain cases [2,12]. The most relevant differential diagnoses in routine clinical practice are summarized in Table 2.

## 3. Pathophysiology

**Figure 1:**
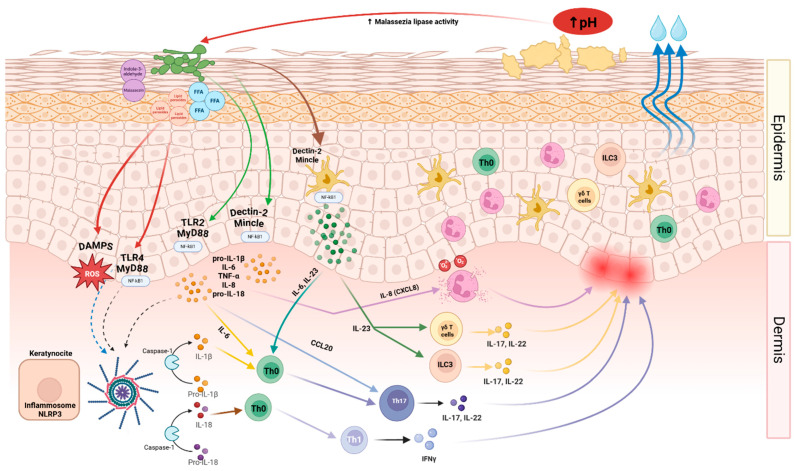
Pathophysiological mechanisms of seborrheic dermatitis. Increased skin pH enhances *Malassezia* lipase activity, leading to the release of free fatty acids (FFA), lipid peroxides, and indole derivatives, including indole-3-aldehyde and malassezin. These metabolites act as danger-associated molecular patterns (DAMPs), triggering innate immune responses. Keratinocytes recognize *Malassezia* products via TLR2/MyD88 and DAMPs via TLR4/MyD88, resulting in NF-κB1 activation and transcription of proinflammatory cytokines (pro-IL-1β, IL-6, TNF-α, IL-8, and pro-IL-18). Concurrently, oxidative stress activates the NLRP3 inflammasome, with caspase-1 processing pro-IL-1β and pro-IL-18 into their active forms. Langerhans cells sense *Malassezia* through Dectin-2/Mincle, producing IL-23 that, together with IL-6, drives Th17 polarization, whereas IL-18 promotes Th1 differentiation. Th17 cells secrete IL-17A/F and IL-22, leading to epidermal hyperplasia and antimicrobial peptide production, while Th1 cells release IFN-γ. Keratinocyte-derived CCL20 recruits additional Th17 cells, and IL-8 promotes neutrophil chemotaxis. Innate lymphoid cells type 3 (ILC3) and γδ T cells provide early sources of IL-17/IL-22, reinforcing inflammation. Altogether, this complex network of fungal metabolites, innate and adaptive immune responses, and keratinocyte dysfunction explains the characteristic erythema and greasy scaling of seborrheic dermatitis. Adapted and summarized from Wikramanayake et al. [9], Adalsteinsson et al. [14], and Sparber et al. [15].

The understanding of the mechanisms involved in SD continues to evolve. The current understanding of seborrheic dermatitis recognizes three major pillars in its pathophysiology: alteration of the skin microbiome, dysfunction of sebaceous gland secretion, and the host immune response (Figure 1). A potential fourth factor is genetic predisposition, although its clinical relevance has not yet been fully established.

### 3.1. Microbiome

SD has been closely linked to alterations in the skin microbiome, particularly involving *Malassezia* spp. [1]. This genus includes more than 14 species of lipophilic yeasts, with *M. globosa* and *M. restricta* being the species most frequently associated with seborrheic dermatitis. Other species described include *M. furfur*, *M. sympodialis*, *M. obtusa*, *M. slooffiae*, and the more recently identified *M. arunalkei*. Species prevalence appears to vary by geographic region [1].

Sex does not seem to influence susceptibility to specific species. However, age plays a key role: *M. globosa* predominates in individuals under 14 years old, while *M. sympodialis* is more prevalent in adults. Anatomically, *M. globosa* is more abundant on the trunk, whereas *M. restricta* predominates on the scalp and forehead [15,16].

The virulence of *Malassezia* is attributed to its lipid-rich cell wall, which provides mechanical stability, osmotic resistance, and protection from phagocytosis [17]. However, the correlation between fungal load and disease severity remains controversial [18].

According to Wikramanayake et al. [9], *Malassezia* spp. proliferation may result from dysbiosis secondary to epidermal barrier dysfunction. Its metabolites, such as oleic acid, penetrate the stratum corneum and trigger a local inflammatory immune response, mediated by proinflammatory cytokines. This microenvironment perpetuates inflammation and barrier impairment (Figure 1).

Tanaka et al. [19] reported increased relative abundance of *Acinetobacter*, *Staphylococcus*, and *Streptococcus* species in lesional SD skin. Other studies highlighted increased colonization by *S. epidermidis* and *S. aureus*, suggesting a role in the cutaneous dysbiosis of SD [20]. Park et al. observed that patients with scalp SD harbored higher levels of *Staphylococcus* spp. and *M. restricta* compared with healthy controls, who instead showed higher levels of *Propionibacterium* spp. and *M. globosa* [21]. The imbalance between fungal and bacterial communities appears to be a key driver in SD pathophysiology.

### 3.2. Sebum Secretion

Sebaceous glands are holocrine structures regulated by androgens and cortisol derived from the adrenal glands. They are distributed throughout the body, with the highest density on the face and upper trunk, excluding palms and soles [22]. Under cutaneous homeostasis, both keratinocytes and sebocytes contribute to the production of lipids. Keratinocyte-derived lipids integrate into the stratum corneum, while sebocyte-derived lipids are secreted onto the skin surface. Squalene serves as a lipid biomarker to distinguish sebaceous from epidermal lipid origin [23].

*Malassezia* spp. produces enzymes such as lipases and phosphatases that hydrolyze sebaceous lipids [24]. This process reduces triglyceride levels and increases free fatty acids. The yeast preferentially consumes saturated fatty acids, leaving behind unsaturated fatty acids (UFAs). Among these, oleic acid is particularly relevant, as it penetrates the stratum corneum, promotes inflammation, and induces desquamation, thereby exacerbating clinical features of SD such as flaking [25].

### 3.3. Immune System and Skin Barrier

*Malassezia* interacts directly with the innate immune system. It promotes dendritic cell maturation and inflammasome activation through multiple inflammatory cascades. It also stimulates keratinocytes and antigen-presenting cells via pattern recognition receptors such as Toll-like receptors (TLR2), NOD-like receptors (NLRs), and C-type lectin receptors [1].

Scientific advances have expanded current knowledge of the inflammatory pathways and barrier abnormalities underlying SD. Altered expression of keratins K1, K10, and K11 has been observed [8], along with reduced levels of ceramides and sphingolipids [26].

Among the most relevant pattern recognition receptors (PRRs) are C-type lectin receptors, particularly Dectin-2, which recognize Malassezia cell wall components and activate the SYK–CARD9 signaling pathway [27]. This leads to dendritic cell maturation, phagocytosis, and secretion of proinflammatory cytokines such as IL-6 and IL-23, ultimately driving Th17/Th22 polarization, hallmarks of chronic skin inflammation [8].

Keratinocytes detect *Malassezia* spp. via TLR2, triggering MyD88/NF-κB signaling and promoting the release of IL-8, IL-6, and TNF-α, thereby enhancing neutrophil and lymphocyte recruitment [28]. *M. globosa* and *M. restricta* appear to be especially potent in stimulating TLR2, supporting their predominant pathogenic role in SD [29]. Additionally, NOD-like receptors (NLRs), especially NLRP3, become activated, leading to inflammasome assembly, caspase-1 activation, and conversion of pro–IL-1β into its active form [30], amplifying the release of IL-1β and IL-18, which further supports the Th1, Th17/Th22, Th2 axis [30,31,32] (Figure 1).

Elevated levels of multiple inflammatory cytokines, including IL-1α, TNF-α, IFN-γ, IL-6, IL-17, IL-18, and IL-23, have been reported in lesional biopsies from patients with seborrheic dermatitis compared with healthy skin from volunteers, indicating simultaneous activation of Th1, Th2, and Th17 pathways [33]. Moreover, increased IL-17–producing γδ T cells in Mpzl3 knockout mice (as a model for SD) further support a central role for the IL-17 axis in SD pathogenesis [34].

Taken together, seborrheic dermatitis is a model of chronic skin inflammation driven by the interplay between dysbiosis, impaired barrier function, and innate immune activation, with *Malassezia* acting as a key orchestrator of this response.

Overall, the current evidence suggests that *Malassezia* acts as a metabolic and immunologic catalyst rather than a simple commensal. Through lipid hydrolysis and the production of bioactive metabolites, these yeasts alter the cutaneous microenvironment, compromise the epidermal barrier, and perpetuate inflammation by promoting Th17/Th22 polarization. This integrated view links microbiome dysbiosis, barrier dysfunction, and immune activation, establishing *Malassezia*-driven immunometabolic imbalance as a central pathogenic axis in SD.

### 3.4. Genetic Predisposition

The role of genetic predisposition in SD remains uncertain. Associations with specific HLA alleles and mutations in genes involved in cutaneous immunity (e.g., *ACT1*, *C5*, *IKBKG*, *STK4*) and epidermal differentiation (*ZNF750*) have been proposed [35]. However, genome-wide association studies (GWAS) have not identified consistent risk *loci*, except for the *LCE3* gene cluster, which is also implicated in psoriasis and atopic dermatitis [14]. While a genetic contribution to SD pathogenesis is plausible, current evidence is still limited and inconclusive.

## 4. Triggering and Exacerbating Factors

DS is influenced by multiple risk factors [2,9,36]. Viral infections (HIV, HCV, SARS-CoV-2), neurological disorders such as Parkinson’s disease, and psychological stress have all been associated with increased disease susceptibility. Hormonal imbalances, particularly those involving androgens, stimulate sebaceous gland activity and alter sebum composition, promoting *Malassezia* proliferation and exacerbating inflammation. Male sex is also a recognized risk factor [2].

Climatic conditions influence disease expression: cold, dry weather exacerbates scaling and pruritus, whereas hot, humid environments promote sweating and fungal overgrowth, contributing to seasonal flares [37]. Dietary factors, including high intake of refined sugars, dairy products, and saturated fats, may impair immune function and disrupt skin microbiota [38].

**Figure 2:**
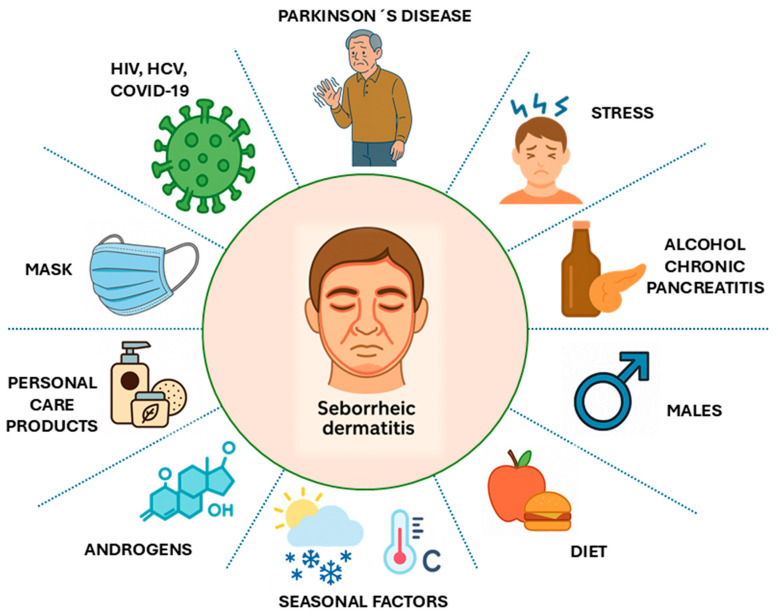
Main factors associated with seborrheic dermatitis. Adapted and summarized from Rietcheck et al. [4] and Wikramanayake et al. [9].

Additional aggravating factors include alcohol consumption, chronic pancreatitis, prolonged use of face masks, and certain skincare products [37]. Figure 2 summarizes the main factors associated with the onset or exacerbation of SD.

## 5. Diagnosis

### 5.1. Clinical Diagnosis: Current Criteria

The diagnosis of SD remains clinical [39]. Key features include poorly defined erythematous-squamous plaques with greasy or yellowish scaling, typically located on seborrheic areas such as the scalp, face, and upper trunk. The chronic and relapsing course supports the diagnosis in the absence of systemic symptoms [10,11]. As no disease-specific biomarkers have been identified, the diagnosis of seborrheic dermatitis continues to rely primarily on clinical judgment. Skin biopsy is not routinely recommended but may be considered in atypical cases, treatment-resistant forms, or when differentiation from other dermatoses [36], such as psoriasis, lupus erythematosus, or atopic dermatitis, is required.

In patients with darker skin phototypes, particularly individuals of African descent, seborrheic dermatitis may present with more subtle erythema, appearing violaceous or hyperpigmented rather than bright red [40]. Greasy scaling is often less prominent or may be masked by coiled hair in scalp involvement, leading to underrecognition or misdiagnosis. Post-inflammatory hyperpigmentation is also more frequent and may persist beyond active disease. Clinicians should be aware of these variations to avoid diagnostic delay and optimize management strategies in patients of color [40].

Ocular involvement is an underrecognized yet clinically relevant manifestation of seborrheic dermatitis, with prevalence estimates ranging from 10% to 40% of affected patients [41]. The high density of sebaceous glands in the periocular region facilitates the proliferation of Malassezia spp. and inflammation, which may extend to the ocular surface. This may lead to blepharitis, conjunctivitis, keratitis, or dry eye disease. Early diagnosis through slit-lamp examination, combined with interdisciplinary management involving dermatologists and ophthalmologists, is essential to prevent complications. Management includes lid hygiene with warm compresses and gentle cleansing, artificial tears for dry eye, and short courses of topical antibiotics or corticosteroids for blepharitis when indicated. In refractory cases, calcineurin inhibitors such as tacrolimus 0.03% ointment, preferably in an ophthalmic or low-irritant formulation, can be used on the eyelids under ophthalmologic supervision [41].

### 5.2. Dermoscopy

Dermoscopy is a valuable tool for the differential diagnosis of SD, particularly in scalp involvement. Typical findings include arborizing vessels, atypical red vessels, featureless areas, and fine white or yellowish scales diffusely distributed over an erythematous background [42].

These dermoscopic features help distinguish SD from other scaly erythematous dermatoses [43]. In scalp psoriasis, for example, scales tend to be thicker, and glomerular vessels and red dots are more commonly observed [44].

### 5.3. Histopathological Findings and Their Use in Atypical Cases

The histopathological features of seborrheic dermatitis are not disease-specific and therefore require correlation with clinical findings for accurate diagnosis. Key features include focal parakeratosis, accumulation of neutrophilic scale at dilated follicular openings, psoriasiform acanthosis, and mild to moderate spongiosis. In the dermis, a predominantly perivascular lymphocytic infiltrate is observed, occasionally accompanied by dendritic cells in the epidermis and perifollicular dermis [45]. In chronic stages, SD may histologically overlap with psoriasis. However, the presence of even subtle spongiosis supports the diagnosis of SD.

In patients with HIV infection, more pronounced histological changes may be observed, correlating with the degree of immunosuppression [46]. These include extensive parakeratosis, reduced spongiosis, dense dermal infiltrate with distortion of the dermoepidermal junction, prominent plasma cells, and leukocytoclasia. These features can aid diagnosis in complex or atypical presentations [47]. In all cases, biopsy should be reserved for selected scenarios and does not replace expert clinical assessment.

### 5.4. Emerging Biomarkers and the Role of Tape Stripping

Validated biomarkers for SD are currently lacking, but several research lines offer promising insights. Elevated serum levels of Raftlin and 8-iso-prostaglandin F2α have been reported in SD [48], suggesting systemic inflammation and oxidative stress. However, their specificity remains limited.

More recently, transcriptomic analysis using non-invasive tape stripping has enabled the molecular profiling of SD. This technique collects superficial epidermal layers through adhesive discs and allows gene expression analysis. Findings include overexpression of cytokines related to the IL-23/Th17 and Th22 pathways (IL23A, IL22, PI3, LL37, S100A8, S100A12), as well as Th1-associated genes (OASL, STAT1, CXCL9), with minimal Th2 involvement [8]. Simultaneously, a downregulation of epidermal barrier markers such as CLDN1, CLDN8, FA2H, and ELOVL3 was observed, suggesting impaired barrier integrity [8]. These molecular patterns may serve as future diagnostic or therapeutic biomarkers, especially through minimally invasive tools like tape stripping.

## 6. Conventional Treatment

Management of seborrheic dermatitis focuses on three main targets: controlling inflammation, reducing Malassezia load, and restoring skin barrier function. Given the chronic, relapsing course of the disease, treatment should be tailored to the affected site, disease severity, and individual patient characteristics. Topical antifungals remain the first-line therapy [49]. Combination with corticosteroids, topical calcineurin inhibitors (TCIs), keratolytics, or targeted dermocosmetic products often enhances clinical control and reduces adverse effects [50]. In particular, TCIs such as pimecrolimus and tacrolimus represent a valuable non-steroidal alternative for the treatment of facial seborrheic dermatitis, especially in sensitive areas and in patients who are corticosteroid-phobic [51]. These agents inhibit T-cell activation and downstream pro-inflammatory cytokine production without causing skin atrophy. Clinical studies have demonstrated significant improvement within two weeks of treatment, with reduced severity upon recurrence. Although their use may cause mild burning or irritation, long-term safety data, primarily derived from AD, support their favorable safety profile, including no evidence of systemic immunosuppression or increased malignancy risk [52]. In patients with severe seborrhea or refractory facial and scalp involvement, low-dose oral isotretinoin (e.g., 10 mg every other day) may be considered for its sebosuppressive and anti-inflammatory effects [53]. In refractory cases, short courses of oral antifungals such as fluconazole [54] or itraconazole [55] may be considered.

Treatment selection depends on anatomical site (scalp, face, trunk) and flare frequency. Table 3 summarizes commonly used therapies according to anatomical site and line of treatment.

## 7. Emerging Therapies and Future Perspectives

Although conventional topical therapies offer satisfactory control in most cases of SD, frequent relapses, limited tolerability, and the lack of approved treatments for this specific indication highlight the need for novel therapeutic strategies.

### 7.1. Phosphodiesterase 4 (PDE4) Inhibitors

PDE4 inhibitors are an emerging therapeutic class in dermatology, offering targeted control of inflammation in several chronic dermatoses [56]. While apremilast (oral) and crisaborole (topical) have been approved for psoriasis and atopic dermatitis, respectively, roflumilast is the first topical PDE4 inhibitor approved for SD, with demonstrated efficacy and favorable safety in clinical trials [57,58]. PDE4 is an enzyme that degrades cyclic adenosine monophosphate (cAMP), a key regulator of the inflammatory response. Its inhibition increases intracellular cAMP levels, thereby suppressing proinflammatory cytokines (IL-2, IL-4, IL-17, IL-23, TNF-α) and enhancing IL-10, an anti-inflammatory cytokine [59]. This mechanism is particularly relevant in seborrheic dermatitis, where Th1, Th17, and Th22 immune responses converge with *Malassezia*-driven inflammation and barrier dysfunction. Moreover, PDE4 inhibition may help restore a more balanced immune microenvironment, potentially influencing the skin microbiome, although this effect is indirect [58].

Three PDE4 inhibitors have shown potential in the treatment of seborrheic dermatitis:

- Roflumilast 0.3% foam or cream, a selective PDE4 inhibitor initially approved for plaque psoriasis, has recently demonstrated promising results in SD. In a recent phase IIa clinical trial, 73.8% of patients treated with roflumilast foam (0.3%) achieved Investigator Global Assessment (IGA) success at week 8, compared with 40.9% in the vehicle group (*p* < 0.001) [60]. These findings were confirmed in a phase III trial, in which 79.5% of patients treated with roflumilast cream achieved IGA success compared with 58.0% in the vehicle group (*p* < 0.001) [58,61]. Significant reductions in erythema, scaling, and itch severity were also observed. Importantly, roflumilast showed a favorable safety profile, with adverse events comparable to vehicle foam. Unlike topical corticosteroids or calcineurin inhibitors, roflumilast is non-steroidal, lipophilic, and formulated for once-daily use, making it especially attractive for visible areas such as the face and scalp. Its cosmetic acceptability, low irritation potential, and anti-inflammatory efficacy support its clinical utility in chronic, relapsing SD.
- Crisaborole 2% ointment, indicated for mild-to-moderate atopic dermatitis, also inhibits PDE4 and may be beneficial for facial SD or sensitive skin [62]. Clinical experience is limited to anecdotal reports. Its high cost may restrict broader use.
- Apremilast, an oral PDE4 inhibitor approved for psoriasis and psoriatic arthritis, has shown potential in the management of SD, particularly in isolated, recalcitrant cases [63].

### 7.2. Biologic Therapies

Biologic therapies are not approved for the treatment of SD and are reserved for exceptional circumstances. Nevertheless, clinical observations in patients receiving IL-17 inhibitors (e.g., secukinumab) or IL-23 inhibitors (e.g., guselkumab) for comorbid psoriasis have reported concomitant improvement in seborrheic dermatitis, supporting a potential contribution of the Th17 axis in selected patient subgroups [64]. In addition, a small case series described off-label use of ustekinumab, where five of six patients achieved complete clinical response after three to five doses, suggesting a possible benefit of IL-12/23 blockade in refractory cases; however, evidence remains anecdotal and insufficient to inform clinical practice [65].

In contrast, dupilumab, an IL-4Rα antagonist that blocks IL-4 and IL-13, has shown controversial results in SD. While some anecdotal reports describe improvement in facial SD in patients treated for atopic dermatitis, growing evidence suggests that dupilumab may induce or worsen seborrheic-like lesions [66]. This paradoxical reaction typically affects sebaceous-rich facial areas. The proposed mechanism involves a local immune imbalance, with compensatory Th17 dominance after Th2 pathway inhibition [67].

### 7.3. JAK Inhibitors

The JAK/STAT pathway regulates intracellular signaling of multiple cytokines involved in inflammatory dermatoses, including IL-4, IL-13, IL-6, IFN-γ, IL-31, and Thymic Stromal Lymphopoietin (TSLP). Inhibiting this pathway allows modulation of Th1, Th2, and Th17 responses simultaneously [68], making it particularly attractive in diseases with mixed immunologic profiles such as SD.

Ruxolitinib cream 1.5%, a topical JAK1/JAK2 inhibitor approved for mild-to-moderate atopic dermatitis and vitiligo, has shown a favorable pharmacological profile for facial SD. With its rapid onset, favorable safety profile in sensitive areas, and absence of rebound effects, it emerges as a promising therapeutic option for chronic or refractory facial seborrheic dermatitis, although further controlled studies are warranted. Studies in other inflammatory dermatoses confirm their ability to reduce erythema, pruritus, and barrier dysfunction without inducing atrophy or sensitization. Although not formally approved for SD, off-label use may be justified in selected cases [69,70]. A case report described a favorable response to topical ruxolitinib 1.5% in a patient with seborrheic dermatitis associated with rosacea, supporting the potential role of JAK inhibition in mixed or refractory inflammatory phenotypes; however, evidence remains anecdotal [69].

Delgocitinib, a topical pan-JAK inhibitor approved in Japan for atopic dermatitis, also appears promising for facial SD [71]. Its broad immunomodulatory activity and favorable safety profile make it an attractive option for corticosteroid-averse patients or those with highly reactive skin. Beyond seborrheic dermatitis, topical delgocitinib is currently under investigation for other indications, including chronic hand eczema and inverse psoriasis [72,73].

Oral JAK inhibitors (e.g., upadacitinib, abrocitinib, baricitinib) have emerged as effective oral therapies for atopic dermatitis (AD), including in patients with overlapping AD/SD features [74]. Their broad inhibition of cytokines (IL-4, IL-13, IL-31, IL-22, and IFN-γ) contributes to rapid control of inflammation, especially in difficult-to-treat facial and scalp areas [75].

Recent case reports have described paradoxical seborrheic dermatitis–like eruptions induced by JAK inhibitors, particularly upadacitinib, in patients who initially responded well to treatment. In one report, a 35-year-old male developed a crusted seborrheic-pattern dermatitis after six months of therapy, despite prior improvement in atopic dermatitis [76]. This reaction may reflect a shift toward Th1/Th17-driven inflammation. Although uncommon, these observations underscore the importance of careful monitoring and individualized management in AD patients with prominent facial involvement receiving JAK inhibitors. Nevertheless, their efficacy in overlapping AD/SD phenotypes remains promising in selected and refractory cases [12].

Overall, topical and systemic JAK inhibitors represent an innovative therapeutic avenue for seborrheic dermatitis. They may be particularly valuable in refractory cases, in patients intolerant to corticosteroids, or in those with overlapping inflammatory dermatoses [77].

### 7.4. Topical Probiotics and Microbiome-Targeted Therapies

The relationship between probiotics and seborrheic dermatitis is primarily mediated through modulation of the cutaneous microbiome and, to a lesser extent, the gut–skin axis. SD is characterized by a dysbiotic profile with increased abundance of *Malassezia* spp. [1]. and *Staphylococcus* spp., accompanied by reduced levels of *Cutibacterium acnes* and *Lactobacillus* spp. in affected skin. This microbial imbalance is thought to contribute to persistent inflammation and impaired skin barrier function [78].

Recent studies have demonstrated that topical application of specific probiotics, including *Lactobacillus crispatus* and *Lacticaseibacillus paracasei*, can reduce disease severity while promoting a more favorable cutaneous microbiome composition [79]. Reported improvements include a decreased abundance of *Malassezia* and *Staphylococcus* spp., together with an increase in beneficial commensals such as *Lactobacillus*. Beyond live probiotics, postbiotics (bacterial lysates or immunomodulatory metabolites) and prebiotics (substrates that enhance the growth of beneficial microorganisms) are also emerging as well-tolerated alternatives to support long-term restoration of the skin ecosystem [80].

Although clinical evidence remains limited, preliminary findings suggest potential benefits of probiotics in alleviating symptoms and restoring microbial balance [81]. Systematic reviews and interventional studies have reported that both oral and topical formulations can modulate skin immune responses, enhance barrier integrity, and reduce inflammation. Most of these observations, however, derive from studies in atopic dermatitis rather than seborrheic dermatitis [81].

At present, no dermatological guidelines recommend the routine use of probiotics for SD, and their application should be considered experimental and individualized until high-quality, long-term randomized controlled trials are available. Nevertheless, growing interest in the microbiome as a therapeutic target suggests that microbiome-directed interventions may evolve into valuable adjuncts or alternatives to conventional antifungals, particularly in patients with recurrent disease, sensitive skin, or corticosteroid aversion [82].

### 7.5. Skin Barrier Modulation

Skin barrier repair is an emerging therapeutic focus in SD, particularly for facial involvement and recurrent flares. Increased transepidermal water loss (TEWL), altered lipid composition with reduced ceramide content, alkalinization of the skin surface pH, and disrupted expression of structural proteins contribute to inflammation and Malassezia overgrowth [26] (Figure 1). Although evidence in SD is limited, data from AD support the use of barrier-enhancing emollients containing ceramides, cholesterol, linoleic acid, and niacinamide to restore epidermal integrity and reduce inflammation [83]. Additionally, topical prebiotics and microbiome-friendly formulations may contribute to rebalancing the cutaneous flora, although their role in seborrheic dermatitis remains largely theoretical [80]. As adjunctive therapies, they may enhance tolerability to antifungals or calcineurin inhibitors and support long-term remission in corticosteroid-sensitive areas.

Despite recent advances in understanding seborrheic dermatitis, the mechanisms governing remission and relapse remain largely unclear. It is still unknown whether disease recurrence results mainly from persistent *Malassezia* colonization, residual immune activation, or incomplete restoration of the skin barrier. Moreover, the relative contribution of environmental triggers, host susceptibility, and microbiome dynamics during remission is poorly characterized. Longitudinal, multi-omics studies integrating clinical, microbial, and immunological data are needed to elucidate these mechanisms and identify biomarkers capable of predicting disease recurrence or sustained remission.

## 8. Conclusions and Future Directions

Despite the efficacy of conventional therapies, a subset of patients with seborrheic dermatitis continues to experience refractory, relapsing, or persistent facial involvement. In this context, emerging treatments provide an opportunity to target the underlying pathophysiological mechanisms and achieve more durable disease control. Their further development and validation in well-designed clinical studies will be essential to advance toward more precise and personalized management of this chronic inflammatory skin disorder.

## Figures and Tables

**Table 1:** Clinical Features and Atypical Forms of Seborrheic Dermatitis by Age Group [2,12]. Abbreviations: SD, seborrheic dermatitis; AD, atopic dermatitis.

Age Group	Prevalence and Associated Factors	Typical Locations	Main Clinical Features	Atypical Forms/Clinical Variants	Comments/Clinical Observations
Infants (0–12 months)	Up to 70%. Maternal androgen influence	Scalp (“cradle cap”), eyebrows, eyelids, retroauricular areas, neck, diaper area	Adherent, yellowish, greasy, asymptomatic plaques	Leiner’s disease (severe erythrodermic variant)	Self-limited. Resolves within weeks/months. No pruritus
Children (2–12 years)	Low prevalence. Reduced sebaceous activity	Scalp, retroauricular areas, mild facial involvement	Fine scaling, mild dermatitis in seborrheic areas	Impetiginization. Mild perioral form	May be mistaken for AD or impetigo. Often underdiagnosed
Adolescents (13–18 years)	Up to 8%. Increased pubertal androgens	Facial T-zone, scalp, upper chest	Erythema, greasy scaling. Often confused with dandruff or seborrheic acne	Papulosquamous thoracic form. SD resistant and associated with acne	Hormonal influence relevant. Mild-to-moderate course
Young/Middle-aged Adults (20–50 years)	2–5%. Relapses triggered by stress, climate, irritants	Scalp, eyebrows, nasolabial folds, glabella, retroauricular areas, upper chest	Erythematous-scaling plaques. Yellowish, oily scales. Frequent pruritus	Sebopsoriasis. Extensive facial form. Rosacea-like	Chronic and relapsing course. Higher prevalence in men and stressed individuals
Older Adults (>60 years)	3–10%. More common in neurological comorbidities, immunosuppression	Scalp, face, external auditory canal, folds	More inflammatory, scaly, pruritic lesions. May mimic erythroderma	Erythrodermic SD. Hypopigmented macules. SD associated with Parkinson’s disease	Lower treatment response. Frequent comorbidities. May coexist with other dermatoses
Immunosuppressed (HIV, lymphoma, etc.)	High prevalence in those with HIV (up to 78%). Early cutaneous marker	Central facial region, axillae, groin, trunk	Extensive, inflammatory, scaly lesions with severe pruritus	Refractory form. Generalized SD. Intense seborrhea	Requires oral antifungals and immunomodulators. Limited response to standard treatment

**Table 2:** Differential Diagnosis of Seborrheic Dermatitis (SD): Key Clinical and Diagnostic Features. Abbreviations: SD, seborrheic dermatitis; ANA, antinuclear antibodies; VDRL, Venereal Disease Research Laboratory test; TPHA, Treponema pallidum hemagglutination assay; KOH, potassium hydroxide.

Entity	Key Clinical Features	Differences from SD	Affected Areas	Diagnostic Clues and Comments
Psoriasis vulgaris	Well-demarcated erythematous plaques with dry, white or silvery scales. Often extends beyond the hairline (“corona sign”)	Drier, thicker scales; sharply demarcated plaques. Nail and extensor area involvement is common	Scalp, elbows, knees, sacral area, nails	May coexist with SD (“Sebopsoriasis”). Nail and extensor involvement suggests psoriasis. Histology or treatment response may aid diagnosis
Atopic dermatitis	Chronic eczema, intense pruritus, xerosis. Lichenified or exudative lesions. Often starts in childhood	More severe pruritus. Flexural distribution. History of personal or family atopy. Less seborrhea	Face, neck, folds, trunk	May coexist with SD. Immunologically distinct (Th2 predominance). Distribution and history guide diagnosis
Tinea capitis/corporis	Annular scaly plaques with active borders, often with localized alopecia or broken hairs. More common in children	Well-defined active borders. Does not respond to standard topical antifungals. May present with alopecia	Scalp, face, neck	Diagnosis by KOH prep and fungal culture. In adults, suspect if lesions are unilateral or treatment-resistant
Cutaneous lupus (discoid or subacute)	Erythematous plaques with adherent scale and central atrophy. Photosensitivity. May leave scarring lesions	Marked photosensitivity. Follicular adherent scales. Central atrophy. Permanent lesions	Face, scalp, auricular area	ANA, anti-Ro/SSA may be positive. Skin biopsy and direct immunofluorescence can help confirm the diagnosis. Suspect in refractory cases
Seborrheic rosacea	Persistent centrofacial erythema, telangiectasias, papules/pustules. Scales usually absent	No greasy scaling. Presence of flushing, telangiectasias, inflammatory papules. Limited to face	Nose, cheeks, forehead, chin	May coexist with SD. Flushing and inflammatory lesions support diagnosis. Requires a distinct therapeutic approach
Contact dermatitis (allergic or irritant)	Eczematous pruritic lesions. History of exposure to topical products	Distribution matches contact area. Sudden onset. More exudation or irritation	Face, neck, scalp (often cosmetic-induced)	Confirm with patch testing. Suspect if flares with shampoos or creams
Secondary syphilis	Papulosquamous eruptions, patchy alopecia (“moth-eaten”), mucosal involvement and palmoplantar lesions	Systemic involvement. Generalized distribution. Lesions not greasy. Relevant clinical context	Scalp, trunk, palms, mucosa	Serology (VDRL, TPHA) confirms. Consider in atypical or refractory cases
Folliculitis/impetigo/cellulitis	Pustular or crusted lesions with erythema, pain, and possible fever.	Overt acute infection. No greasy scaling. Rapid evolution	Face, scalp, folds	SD may predispose to secondary infections. SD may predispose to secondary infection; always assess for superinfection in inflamed or exudative lesions

**Table 3:** Topical Treatment of SD according to Anatomical Site [39].

Location	Line of Treatment	Active Ingredient	Concentration and Vehicle	Typical Dosage
Scalp	First-line	Ketoconazole	2% shampoo or gel	2–3 times/week, leave on for 5–10 min
		Ciclopirox olamine	1.5% shampoo	2–3 times/week, leave on for 5–10 min
		Zinc pyrithione *	1% shampoo	2–3 times/week
		Selenium sulfide	2.5% shampoo	2 times/week
	Second-line	Salicylic acid	3–6% solution or shampoo	Twice weekly or alternating with topical antifungals
		Clobetasol propionate	0.05% lotion, foam, or shampoo	Once daily, short cycles (max. 2 weeks)
		Betamethasone valerate	0.1% lotion, foam, or shampoo	Once daily during acute flares
	Maintenance	Ketoconazole or Ciclopirox olamine	2% shampoo or gel 1.5% shampoo	Once weekly
		Emollient shampoos (non-detergent)	—	Frequent use
Face	First-line	Ketoconazole	2% cream or gel	Twice daily until remission
		Ciclopirox olamine	1% cream	Twice daily until remission
		Pimecrolimus	1% cream	Twice daily during flares or maintenance
		Metronidazole	0.75–1% gel or cream	1–2 times/day
	Second-line	Hydrocortisone	1% cream	1–2 times/day for 5–7 days
		Desonide	0.05% gel or cream	1–2 times/day in short flares
	Maintenance	Tacrolimus or pimecrolimus	Tacrolimus 0.03–0.1% ointment Pimecrolimus 1% cream	2–3 times/week, as tolerated
		Seboregulating formulations with niacinamide or zinc	Light cream/emulsion	Daily use
Trunk	First-line	Ketoconazole	2% gel, cream, or shampoo	1–2 times/day
		Ciclopirox olamine	1% cream, gel, or shampoo	1–2 times/day
		Sertaconazole	2% cream	1–2 times/day
	Second-line	Mometasone furoate	0.1% cream	Once daily for 5–7 days
		Betamethasone dipropionate	0.05% cream	Once daily during acute flares
	Maintenance	Ketoconazole, or Ciclopirox olamine	2% gel, cream, or shampoo 1% cream, gel, or shampoo	Twice weekly
		Barrier-restoring emollients	Fragrance-free cream or lotion	1–2 times/day

* Note: Zinc pyrithione was banned in cosmetic products in the European Union in 2022 due to environmental concerns. In regions where this agent is no longer available, alternative antifungal shampoos containing ketoconazole, ciclopirox olamine, or selenium sulfide can be used, offering comparable efficacy and safety profiles.

## Data Availability

Data sharing is not applicable to this article as no new data were created or analyzed in this study.
